# Spatiotemporal Encoding With Nonlinear Gradient Hardware Using Pulseq: From Principles to Practical Demonstration

**DOI:** 10.1002/mrm.70277

**Published:** 2026-02-09

**Authors:** Andreas Holl, Frank Zijlstra, Maxim Zaitsev, Jakob Hufschmidt, Shadi Tashakori, Nils Schallner, Thomas Stieglitz, Jens Gröbner, Sebastian Littin

**Affiliations:** ^1^ Division of Medical Physics, Department of Diagnostic and Interventional Radiology University Medical Center Freiburg, Faculty of Medicine, University of Freiburg Freiburg Germany; ^2^ Department of Radiology and Nuclear Medicine St Olav's University Hospital Trondheim Norway; ^3^ Department of Anesthesiology and Critical Care Medical Center ‐ University of Freiburg, Faculty of Medicine, University of Freiburg Freiburg Germany; ^4^ Laboratory for Biomedical Microtechnology, Department of Microsystems Engineering‐IMTEK & BrainLinks‐BrainTools Center University of Freiburg Freiburg Germany; ^5^ Department of Electrical Engineering & Information Technology South Westphalia University of Applied Sciences Lüdenscheid Germany

**Keywords:** MRI, nonlinear gradient, Pulseq, quadratic phase, spatiotemporal‐encoding, SPEN

## Abstract

**Purpose:**

Provide the theoretical foundation and the first practical demonstration of spatiotemporal encoding (SPEN) using additional nonlinear gradient hardware.

**Methods:**

The quadratic phase profile can be generated either by a chirped‐RF pulse combined with a constant gradient or, directly, by a quadratic gradient pulse. Both a conventional chirped‐RF and a novel SPEN method using a custom‐built matrix gradient coil for quadratic phase generation were implemented and integrated into a spin‐echo echo‐planar‐imaging (SE‐EPI) sequence using Pulseq. The methods were compared through phantom imaging experiments performed on a 3T MRI system.

**Results:**

The required quadratic phase profile for SPEN was successfully generated using the nonlinear gradient coil, resulting in images of comparable quality. This quadratic gradient‐based approach was achieved while exploiting the advantages of SPEN and overcoming current SAR and minimal TE limitations arising from the use of chirped‐RF pulses.

**Conclusion:**

The generation of the SPEN‐defining quadratic phase using nonlinear gradients is an advantageous alternative to conventional methods. This approach enables improved clinical applicability of SPEN, particularly for 3D and high‐field MRI, by mitigating critical safety and timing limitations. Additionally, an implementation of the conventional method is provided open‐source to support further research.

## 1 Introduction

Spatiotemporal encoding (SPEN) is a non‐Fourier magnetic resonance imaging (MRI) technique renowned for its intrinsic robustness against geometric distortions and chemical shift artifacts, which commonly plague fast imaging methods such as single‐shot echo‐planar imaging (EPI) [1, 2, 3, 4, 5]. The central principle of SPEN is the application of a quadratic phase profile across the field of view (FOV), which establishes a direct correspondence between the acquisition time and the spatial position [1].

Conventionally, this essential quadratic phase is generated by applying a linearly frequency‐modulated (chirped) radio frequency (RF) pulse in the presence of a constant magnetic field gradient [1, 6]. Although effective, this approach has significant drawbacks that have hindered the widespread adoption of SPEN in clinical practice. The use of high‐bandwidth, long‐duration chirped‐RF pulses substantially increases the specific absorption rate (SAR), posing a safety concern, especially at higher field strengths. Furthermore, prolonged RF pulse duration increases the minimum achievable echo time (TE) and can introduce sensitivity to B1‐field inhomogeneities. Moreover, the chirped‐RF pulse lacks selectivity in spatial directions perpendicular to the quadratic phase profile, causing undesired saturation effects in multi‐slice (MS) imaging.

However, the physical principle of SPEN is only dependent on the presence of the quadratic phase, not on the specific method of its generation. This opens a door to alternative implementations that could circumvent drawbacks of the conventional approach. Theoretically, a similar quadratic phase profile can be induced directly by spatial encoding magnetic fields (SEMs) that vary quadratically with a spatial position. Quadratic encoding fields have been proposed to accelerate MR image acquisition in applications such as PatLoc imaging [7, 8] and O‐space imaging [9] and the principle of using quadratic gradients for SPEN has previously been proposed and demonstrated using static second‐order shim coils [10]. However, implementing the latter approach is challenging due to the lack of precise temporal control of the active shim coils and the fact that these coils are typically not magnetically shielded [10].

In this study, we demonstrate that the use of additional nonlinear gradient hardware can successfully generate the desired SPEN within a spin‐echo EPI sequence. We show that it not only addresses the primary drawbacks of the conventional RF‐based technique by significantly reducing SAR and permitting shorter TEs, but also provides a more viable and flexible route towards efficient MS SPEN imaging. Such an approach would decouple SPEN from the RF pulse, thereby eliminating the associated constraints on the SAR pulse duration, and further mitigate out‐of‐slice saturation. Although the idea of using nonlinear gradients for spatial encoding is not new [7, 9, 10], its application as a direct replacement for the chirped‐RF pulse in SPEN has not been practically demonstrated.

The purpose of this work is two‐fold. First, we establish a theoretical framework for SPEN from a forward‐operator perspective, demonstrating that the required quadratic phase profile can be generated either by a conventional chirped‐RF pulse in the presence of a constant gradient or directly by applying a quadratic SEM. Second, we present the first experimental realization and validation of this concept using a custom‐built matrix gradient system. We compare this novel implementation directly to the conventional chirped‐RF‐based SPEN approach, confirming that the quadratic gradient‐based method not only functions as intended, but also overcomes key limitations in SPEN.

To support reproducibility, promote further development, and provide deeper theoretical insight into SPEN from a forward‐operator perspective, we provide an open‐source implementation of the conventional chirped‐RF SPEN method. This includes educational MATLAB code with SPEN sequences based on the open‐source Pulseq framework [11] combined with a numerical Bloch simulation.

## 2 Theory

SPEN is an alternative encoding method in MRI with greater robustness to off‐resonance artifacts such as geometric distortions and chemical‐shift misregistration [3, 12]. It is particularly beneficial for fast imaging techniques, such as single‐shot EPI, which suffer from off‐resonance effects in the low‐bandwidth phase‐encoding direction [13].

### 2.1 Overview and Fundamental Principles of SPEN

In contrast to conventional Fourier transform‐based methods (e.g., EPI), which sample the entire k‐space and then apply an inverse Fourier transform for image reconstruction, SPEN employs a non‐Fourier encoding along one spatial dimension. In the dimension of SPEN, the signal acquired at each time point corresponds directly to a specific spatial location, obviating the need for a Fourier transform along that axis [1, 14].

The key principle of SPEN is to impose a quadratic phase profile on the transverse magnetization and then read out under a decoding gradient so that the stationary phase point (the vertex of the parabola) scans across the field‐of‐view (FOV) [1, 3]. At each instant, only spins near the stationary point contribute constructively to the signal. This process creates a one‐to‐one correlation between the acquisition time and the spatial position. This direct correspondence allows image reconstruction by simple reordering of the time‐domain signal samples, although this naive approach yields images blurred by the method's intrinsic point‐spread function (PSF) [15].

SPEN as a non‐Fourier encoding strategy offers multiple key advantages over conventional Fourier‐based MRI methods. First, by continuously refocusing spins throughout the acquisition period using a spin‐echo or gradient‐echo scheme, SPEN can operate effectively under T 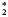
‐free conditions, substantially mitigating susceptibility‐induced artifacts and signal dropouts [12]. Second, the generation of the quadratic phase profile and the formation of an instantaneous spin echo at each time point confine signal contributions to a narrowly defined spatial region around the phase‐stationary point. This inherent localization provides exceptional robustness to magnetic field inhomogeneities and chemical shift offsets, which typically cause severe distortions in EPI [12, 13]. These features make SPEN particularly suitable for challenging imaging scenarios, such as diffusion‐weighted studies in areas of large field variation, imaging in the vicinity of metallic implants [16], and rapid functional MRI in regions like the orbitofrontal cortex [16].

### 2.2 Conventional Generation of the Quadratic Phase Profile in SPEN

The quadratic phase profile in SPEN is usually generated by applying a linearly swept (chirped) RF pulse concurrently with a constant gradient G along the encoding axis (here denoted y) [1]. The chirp pulse has an amplitude modulation (e.g., WURST shape [17, 18]) and a frequency sweep of total bandwidth BW over the duration $T_{\text{enc}}$, corresponding to a sweep rate

$$R=\frac{\text{BW}}{T_{\text{enc}}}\;[\mathrm{Hz}/s].$$

In the rotating frame, the instantaneous RF phase is

(1) $$\phi_{1}(t)=\int_{0}^{t}2\pi f(t^{\prime })\;dt^{\prime }=2\pi \left(f_{0}\;t+\frac{1}{2}R\;t^{2}\right),$$

where $f_{0}$ is the initial frequency offset. Under the gradient G, spins at position y resonate at

$$f_{\text{res}}(y)=f_{\text{iso}}+\gamma \;G\;y,$$

with the gyromagnetic ratio γ and the on‐axis Larmor frequency $f_{\text{iso}}$. A spin at position y is excited when the pulse frequency matches its resonance frequency, that is, $f_{0}+R\;t_{y}=f_{\text{res}}(y)$, resulting in an excitation time

$$t_{y}=\frac{\gamma \;G\;y+(f_{\text{iso}}-f_{0})}{R}.$$

By choosing $f_{0}=f_{\text{iso}}$, the excitation time becomes linearly dependent on position: $t_{y}=(\gamma G/R)y$. Substituting this into the RF phase integral $\phi_{1}(t)$, imparts a phase at position y given by [1]

(2) $$\begin{array}{ll} \phi_{\text{enc}}(y) & =2\pi \left(f_{\text{iso}}t_{y}+\frac{1}{2}Rt_{y}^{2}\right)\\ & =2\pi \left(\gamma G\frac{f_{\text{iso}}}{R}y+\frac{1}{2}R{\left(\frac{\gamma G}{R}y\right)}^{2}\right)\\ & =\underset{\text{linear term}}{\underbrace{2\pi \gamma G\frac{f_{\text{iso}}}{R}\;y}}+\underset{\beta \;y^{2}}{\underbrace{\pi \;\frac{\gamma^{2}\;G^{2}}{R}\;y^{2}}}. \end{array}$$

The linear term can be refocused by a gradient lobe [19]. This leaves the pure quadratic encoding phase

(3) $$\phi_{\text{enc}}(y)=\beta \;y^{2},\;\text{where}\;\beta =\pi \;\frac{\gamma^{2}G^{2}}{R}=\pi \;\frac{\gamma^{2}G^{2}T_{\text{enc}}}{\text{BW}}.$$

This quadratic phase is the cornerstone of the SPEN technique [1, 3]. During readout under a decoding gradient $G_{\text{acq}}(t)$, spins accumulate an additional linear phase, resulting in a total phase modulation of

$$\begin{array}{ll} \phi (y,t) & =\beta \;y^{2}+k_{y}(t)\;y,\\ \text{with}\;k_{y}(t) & =\gamma \int_{0}^{t}G_{\text{acq}}(t^{\prime })\;dt^{\prime }. \end{array}$$

The stationary phase approximation dictates that the signal at time t originates primarily from the location $y_{\text{stat}}(t)$ where the spatial derivative of the phase is zero [1, 2]:

(4) $$\frac{\partial \phi (y,t)}{\partial y}=2\beta y+k_{y}(t)=0\;\;⟹\;\;y_{\text{stat}}(t)=-\frac{k_{y}(t)}{2\beta }.$$

As $k_{y}(t)$ evolves linearly over time, the stationary phase position $y_{\text{stat}}(t)$ sweeps across the FOV at a constant speed. The extent of the FOV is determined by the excitation gradient amplitude and the applied frequency sweep bandwidth, according to

(5) $$\text{FOV}=\frac{BW}{\gamma G_{\text{exc}}}.$$

To ensure uniform sampling across the entire FOV, the stationary phase point is initially positioned at one edge of the FOV using a prephasing gradient. Then, it is shifted across the FOV during acquisition using the imaging gradient, which must satisfy [3, 12, 14]

(6) $$|G_{\text{exc}}T_{\text{exc}}|=\int_{0}^{T_{\text{acq}}}G_{\text{acq}}(t)\;dt.$$

### 2.3 From Fourier to SPEN: the Forward Operator Perspective

In conventional MRI employing Fourier encoding (such as EPI), the relationship between the measured *k*‐space data and the underlying spin density is linear and governed by the discrete Fourier transform (DFT). Each line in *k*‐space samples a unique spatial frequency, and the acquired signal can be written as a matrix vector product

(7) $$\begin{array}{l} s=A_{\text{DFT}}\;x+\eta , \end{array}$$

where x is the vectorized spin density, and the matrix elements are

(8) $$\begin{array}{l} A_{n,k}^{\text{DFT}}=\exp(i2\pi k_{n}y_{k})\Delta y, \end{array}$$

corresponding to a purely linear phase modulation. The image reconstruction is then obtained via the inverse DFT.

In contrast, SPEN introduces a fundamentally different encoding strategy. By imparting a spatially dependent quadratic phase onto the spins, the linear relationship of the DFT is generalized. During SPEN acquisition, the signal at the time point $t_{i}$ (or equivalently at the accumulated gradient area $k_{y,i}$) is given by the integral over the spin density of the object ρ(y):

(9) $$s_{i}=\int_{-\text{FOV}/2}^{+\text{FOV}/2}\rho (y)\exp\left(i[\beta y^{2}+k_{y,i}y]\right)dy+\eta_{i},$$

where $\eta_{i}$ represents measurement noise. The discretization on a spatial grid of positions $y_{j}$ yields the linear system

(10) $$s=A_{\text{SPEN}}x+\eta$$

with the elements of the forward operator matrix $A_{\text{SPEN}}$ given by [15, 20]

(11) $$A_{i,j}^{\text{SPEN}}=\exp\left(i[\beta y_{j}^{2}+k_{y,i}y_{j}]\right)\Delta y.$$

**FIGURE 1:**
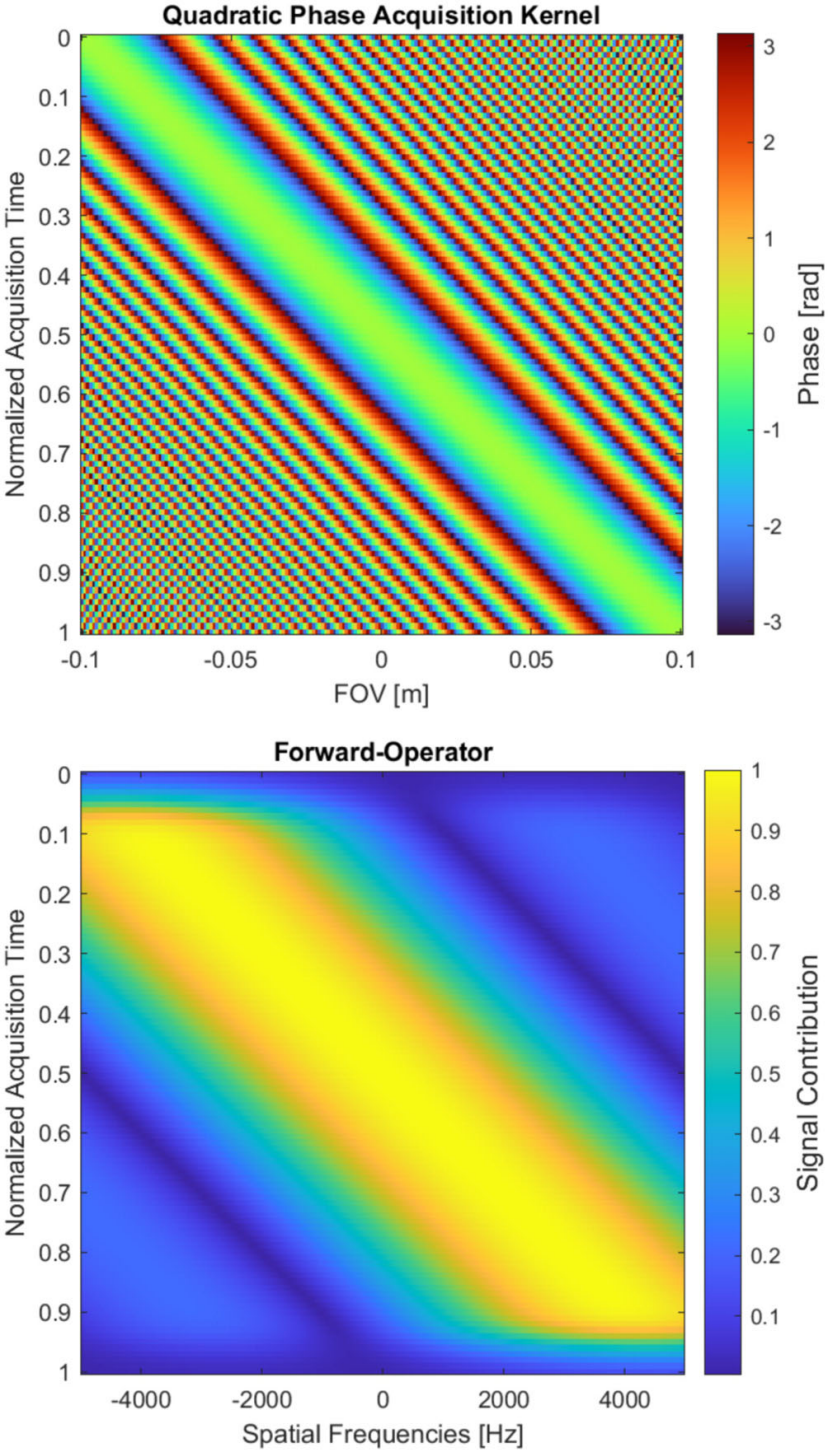
The SPEN mechanism from a spatial perspective and a frequency perspective. (Top) Illustration of the quadratic phase of the SPEN encoding kernel in image domain. Unlike the linear phase ramps of Fourier encoding, SPEN employs a quadratic phase. The signal at each time point is dominated by the contribution from the stationary point (the vertex of the phase parabola), which is swept across the FOV during readout. (Bottom) Visualization of the absolute value of the simulated SPEN forward operator matrix, $A_{\text{SPEN}}$. Each row represents the signal contribution region at a given acquisition time point, depicting the signal localization mechanism of SPEN. The signal contribution decreases drastically at the edges of the FOV because the generation of the quadratic phase profile requires the RF pulse to sweep from far above to far below each spin's resonance frequency. This condition is not met for spins at the very edges of the excitation profile, leading to ineffective excitation and consequently a weaker signal, an effect that is compounded by the inherent excitation profile of the chirped‐RF pulse itself.

Compared to the DFT case, the encoding matrix now contains a quadratic phase term in $y_{j}$. This term breaks the shift‐invariance and global frequency encoding of the DFT and instead establishes a locally focused encoding. The forward operator can be interpreted as a series of time‐varying spatial filters, or lenses, applied to the object. At each acquisition time point $k_{y,i}$, the phase forms a parabola centered at $y_{\text{stat}}(i)=-k_{y,i}/(2\beta )$, which is the stationary phase point. Spins predominately near $y_{\text{stat}}$ contribute coherently to the signal, leading to a localized and direct mapping of time to spatial position, as depicted in Figure 1 [1, 3].

### 2.4 The *k*‐Space Perspective: a Convolution Framework

Although SPEN is often described by its image‐space behavior, it has a clear and powerful interpretation in the frequency domain. This provides the crucial bridge between image‐space and *k*‐space perspectives [21]. The continuous signal equation is as follows:

(12) $$s(k_{y})=\int \rho (y)\cdot \exp(i\beta y^{2})\cdot \exp(ik_{y}y)\;dy$$

This is the Fourier transform of the product of two functions: The spin density ρ(y) and a spatial chirp $\exp(i\beta y^{2})$. According to the Convolution Theorem, the Fourier transform of a product is the convolution of their individual Fourier transforms [19].

**FIGURE 2:**
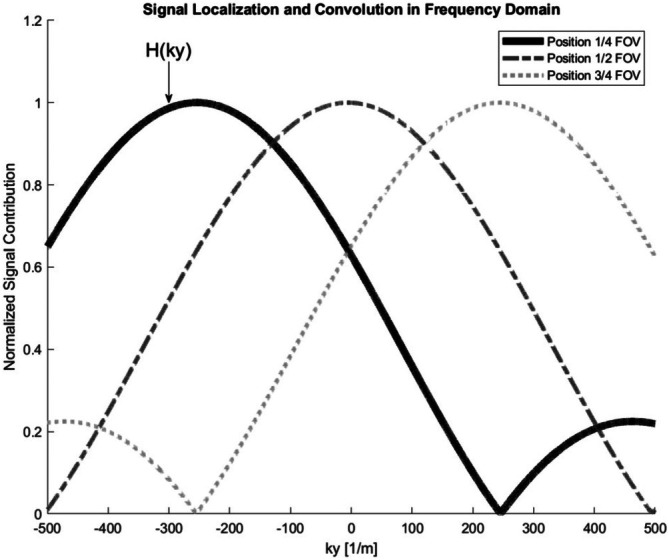
Depiction of the convolution process in the frequency domain. The generation of a quadratic phase profile, $\exp(i\beta y^{2})$, in the image domain results in an equivalent convolution in the frequency domain. According to the Convolution Theorem, the Fourier transform of a product is the convolution of the individual Fourier transforms. The frequency‐space PSF shown, $H(k_{y})$, is the Fourier transform of the spatial quadratic phase. The transform of this spatial chirp is another chirp, $H(k_{y})\propto \exp(-ik_{y}^{2}/(4\beta ))$, whose phase is a parabolic function of the frequency coordinate $k_{y}$. Consequently, the object's true *k*‐space, $P(k_{y})$, is convolved with this quadratic‐phase kernel, resulting in the measured (and inherently smeared) *k*‐space signal $S(k_{y})=P(k_{y})*H(k_{y})$.

Let $P(k_{y})=ℱ\{\rho (y)\}$ be the true *k*‐space representation of the object and $H(k_{y})=ℱ\{\exp(i\beta y^{2})\}$ be the Fourier transform of the spatial chirp. A Fourier transform of a chirp is another chirp, $H(k_{y})\propto \exp(-ik_{y}^{2}/(4\beta ))$ [2, 21]. This function $H(k_{y})$ acts as the *k*‐space point spread function (PSF) or the transfer function of the SPEN encoding, shown in Figure 2.

Therefore, the measured *k*‐space signal, $S(k_{y})$, is the true *k*‐space data, $P(k_{y})$, convolved with the *k*‐space PSF, $H(k_{y})$:

(13) $$S(k_{y})=P(k_{y})*H(k_{y})=\int P(k_{y}^{\prime })H(k_{y}-k_{y}^{\prime })\;dk_{y}^{\prime }$$

This convolution framework is the mathematical bridge: Multiplying by a quadratic phase in the image domain is equivalent to a blurring operation in the *k*‐space domain [21]. Thus, SPEN is fundamentally a *k*‐space encoding method, but one in which data is acquired sequentially in space and inherently smeared or filtered [21]. Image reconstruction then becomes a deconvolution problem in *k*‐space [15, 21].

### 2.5 Information Per Sampled Point: Localized Vs. Global Encoding

The *k*‐space convolution framework allows us to precisely describe the information content of a single sampled point.

- *Conventional Fourier MRI sample*: A single sampled point at $k_{y}$ in a conventional acquisition represents the amplitude and phase of a single sinusoidal wave with spatial frequency $k_{y}$, which extends over the entire field of view. Each point in *k*‐space encodes a global property of the image [22].
- *SPEN MRI sample*: A single sampled point at time t, corresponding to a central *k*‐space value $k_{y}(t)$, does not represent a single frequency. First, it is dominated by the DC signal at the location y(t). Additionally, due to the convolution of the *k*‐space, this sample contains contributions from a range of *k*‐space points around $k_{y}(t)$ [21]. The width of this contribution is determined by the width of the convolution kernel $H(k_{y})$.

In essence, a SPEN acquisition sequentially acquires the local Fourier content of small moving spatial packets from the object, providing a fundamentally different way of sampling image information compared to conventional methods [12].

Effective resolution is governed by the quadratic phase coefficient β. To increase β while maintaining a constant field of view, both the encoding gradient G and the chirp sweep rate R must be proportionally increased [14]. This stronger encoding (a larger β) leads to a more rapid phase dispersion away from the stationary point, effectively narrowing the region of coherent signal contribution and improving the intrinsic resolution and robustness towards off‐resonance effects, at the cost of lower SNR and higher SAR [14, 20]. This trade‐off between resolution, SNR, SAR and robustness towards off‐resonance is a central aspect of the SPEN sequence design.

### 2.6 Alternative Phase Generation Using Quadratic Gradients

As established in the previous sections, the defining characteristic of the SPEN forward operator is the generation of a quadratic phase $\phi (y)=\beta y^{2}$. This single phase term is responsible for the unique dual properties of the encoding: It localizes the signal in the image domain via the stationary phase principle and results in a convolution of the true *k*‐space with a quadratic‐phase kernel $H(k_{y})$ (Equation (13) and Figure 2).

Crucially, the physical consequences of the SPEN encoding only depend on the presence and magnitude of this quadratic phase, not on the specific hardware implementation used to generate it. The conventional method, using a chirped‐RF pulse in combination with a conventional gradient, generates a quadratic coefficient defined in Equation (3). An alternative approach can achieve the exact same initial phase state using a direct quadratic gradient pulse [10]. This method requires specialized hardware to generate a field that varies quadratically with position $B_{\text{quad}}(y)=C\cdot y^{2}$ [23, 24]. Following a short, slice‐selective RF pulse, this gradient is applied for a duration $\tau_{g}$, imparting a phase:

(14) $$\phi (y)=\int_{0}^{\tau_{g}}\gamma B_{\text{quad}}(y)\;dt=\gamma (C\cdot y^{2})\int_{0}^{\tau_{g}}dt=(\gamma C\tau_{g})y^{2}$$

This yields a quadratic coefficient of:

(15) $$\beta_{\text{quad}}=\gamma C\tau_{g}$$

Since both methods can produce an identical initial phase profile, the resulting forward operator $A_{\text{SPEN}}$ is theoretically identical, assuming that the equivalence condition $\beta_{\text{chirp}}=\beta_{\text{quad}}$ is met [10].

However, this theoretical equivalence masks a profound difference in the practical design of the pulse sequence. Although the mathematical goal is the same, the two methods are subject to completely different sets of physical and engineering constraints [10].

- *Chirped‐pulse method*: The magnitude of $\beta_{\text{chirp}}$ is coupled to RF pulse parameters and the linear gradient strength with $\frac{\text{TBW}}{\gamma FOV}=G_{\text{exc}}T_{\text{exc}}=\int_{0}^{T_{\text{acq}}}G_{\text{acq}}(t)\;dt$. Achieving a large $\beta_{\text{quad}}$ for greater robustness toward off‐resonance requires large bandwidth and long‐duration chirped‐RF pulses, leading to a significantly increased SAR. This is a major limiting factor in clinical applications, especially at high fields [10].
- *Quadratic‐gradient method*: The magnitude of $\beta_{\text{quad}}$ is determined solely by the strength (C) of the quadratic gradient and the duration ($\tau_{g}$). This approach decouples phase encoding from RF power deposition, drastically reducing SAR. The limitations here are, instead, the performance of nonlinear gradient hardware (maximum amplitude, slew rate, and field fidelity) [10, 25, 26].

Based on the stationary phase approximation (Equation 4), the robustness to off‐resonances for a given FOV is entirely determined by the maximum duration and amplitude available for both the additional nonlinear gradient and the built‐in linear imaging gradients [8, 10]. This relationship can be expressed as:

(16) $$\begin{array}{l} \Delta k=2\cdot \beta_{\text{quad}}\cdot \text{FOV}=\gamma \int_{0}^{T_{\text{acq}}}G_{\text{acq}}(t)\;dt, \end{array}$$

where Δk is the total coverage of the *k*‐space, $\beta_{\text{quad}}$ is the quadratic phase modulation factor, and $G_{\text{acq}}(t)$ denotes the acquisition gradient over time.

## 3 Methods

We implemented both a SPEN chirped‐RF pulse method and a nonlinear gradient hardware method in the open‐source framework Pulseq [11] and designed one‐dimensional and two‐dimensional SPEN spin‐echo echo‐planar imaging (SPEN‐SE‐EPI) sequences. Both implementations were evaluated using simulations and experiments. As the chirped‐RF SPEN‐SE‐EPI method is non‐selective in the slice dimension, we incorporated our SPEN nonlinear gradient method into a standard SE‐EPI sequence with non‐selective refocusing, in order to create equal conditions for comparing the two SPEN methods. Additionally, we made the chirped‐RF pulse implementation, as well as the educational code for the derivation of the SPEN forward‐operator, publicly available as Open‐SPEN in Pulseq. Furthermore, we included a MS chirped‐RF SPEN‐SE‐EPI version to support the community.

### 3.1 Open‐SPEN Implementation

For the chirped‐RF pulse approach, we implemented a Pulseq‐compatible MATLAB function (MATLAB R2023b, The MathWorks, Natick, MA, USA) to design a wideband, uniform rate, smooth truncation (WURST) chirped‐RF pulse [18] and its corresponding gradient configuration. The excitation gradient amplitude was calculated using Equation (5) to match the spatial frequencies within the desired FOV to the frequency sweep of the RF pulse. The acquisition gradient was configured to satisfy the SPEN condition described in Equation (6).

**FIGURE 3:**
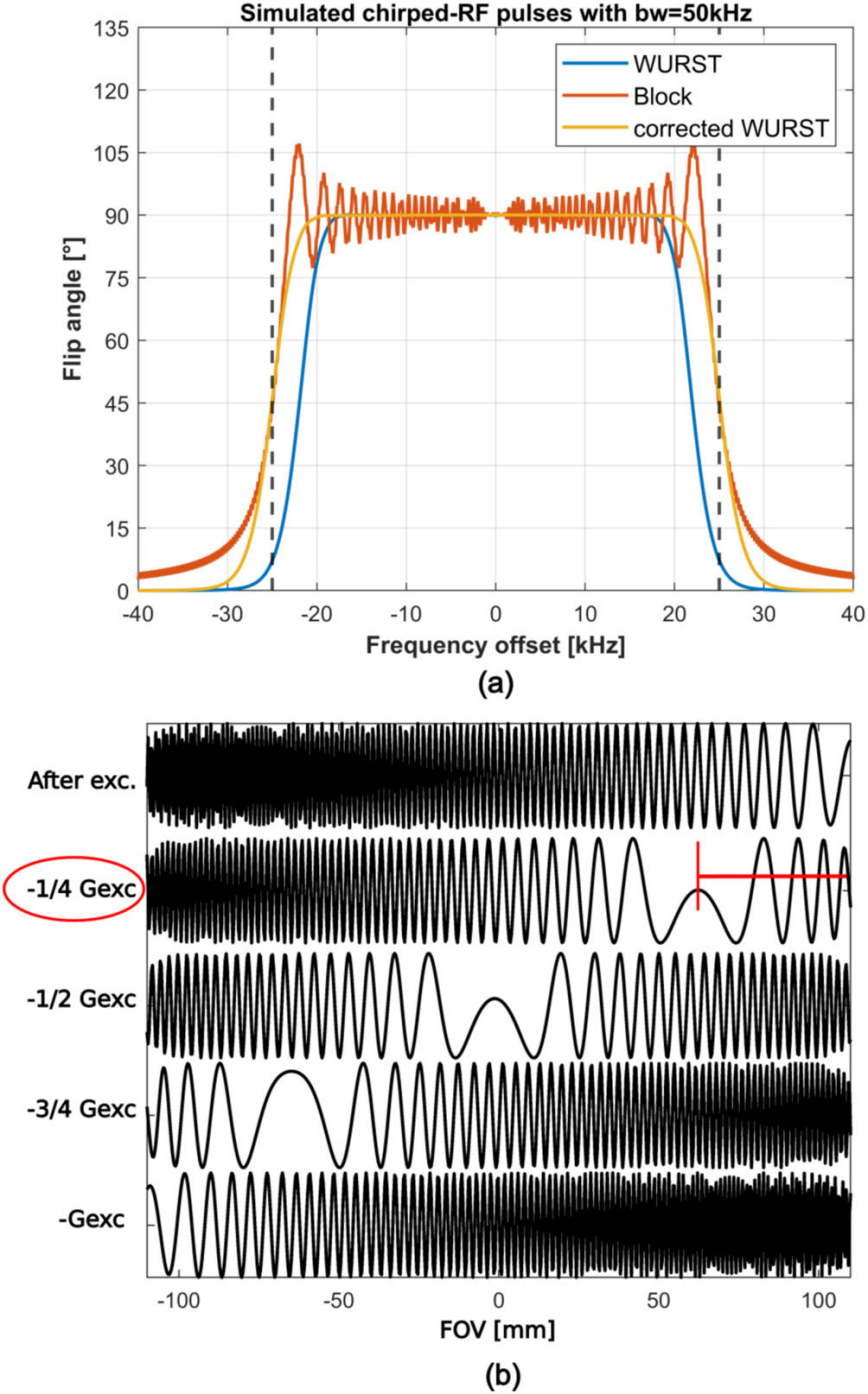
Chirped‐RF pulse and phase simulation results. The simulated flip angle as a function of frequency offset for three chirped‐RF pulses is shown in (a). We simulated a block‐chirped‐RF, an amplitude‐modulated chirped‐RF, and a bandwidth‐corrected chirped‐RF pulse. A sweeping bandwidth of 50 kHz was used, as indicated by the FWHM of the block‐chirped‐RF simulation result. All three chirped‐RF pulses had an RF duration of 4 ms, yielding a time‐bandwidth product (TBW) of 200. The bandwidth‐corrected chirped‐RF pulse smooths the excitation profile while maintaining the desired effective sweeping bandwidth. (b) Depicts the simulated position‐dependent quadratic phase profile after SPEN excitation and during acquisition. A bandwidth‐corrected chirped‐RF pulse was employed for the numerical Bloch simulation in MATLAB, combined with an appropriate encoding gradient to achieve the desired FOV. As shown in (b), a quadratic phase profile can be generated and used for encoding by shifting the vertex of the phase profile across the FOV with appropriately designed acquisition gradient blips.

**FIGURE 4:**
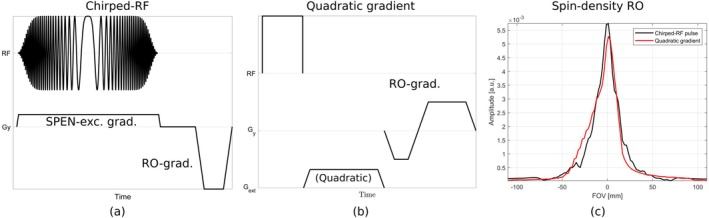
SPEN 1‐D spin‐density read‐out experiment. The SPEN chirped‐RF (a) and SPEN external gradient (b) sequences used for the spin‐density readout experiment are displayed. Both sequences were implemented in Pulseq. For the SPEN chirped‐RF sequence in (a), the Open‐SPEN implementation was used. Both sequences represent the principle of SPEN excitation and acquisition, which can be combined with different encoding methods for multi‐dimensional experiments. The sequence in (a) uses a sweeping bandwidth of 50 kHz, a RF duration of four milliseconds and a gradient‐strength of $250\;\frac{\text{kHz}}{m}$. The external gradient for the desired quadratic phase profile required for the sequence in (b) was fitted in advance (Figure 7a) and then applied to the excited spins before the readout. (c) Shows that with both methods, a direct correlation between the acquisition time and the spin's position is established, making a spin‐density readout of a water bottle phantom possible. For the reconstruction of the spin‐density signal in (c), a convolution‐based reconstruction method was applied to the acquired time signal.

First, we simulated the chirped‐RF pulse using Pulseq's RF simulation environment to evaluate its excitation and phase profile across a range of frequency offsets (Figure 3). We implemented a bandwidth correction to compensate for the reduced width of the excitation profile caused by the amplitude modulation. We then performed a one‐dimensional SPEN excitation and acquisition simulation using our Pulseq function and a numerical Bloch simulation in MATLAB. These simulations were validated with experimental 1D spin density readouts from a water bottle phantom on a clinical MRI scanner. Data were acquired using the experimental sequence shown in Figure 4.

**FIGURE 5:**
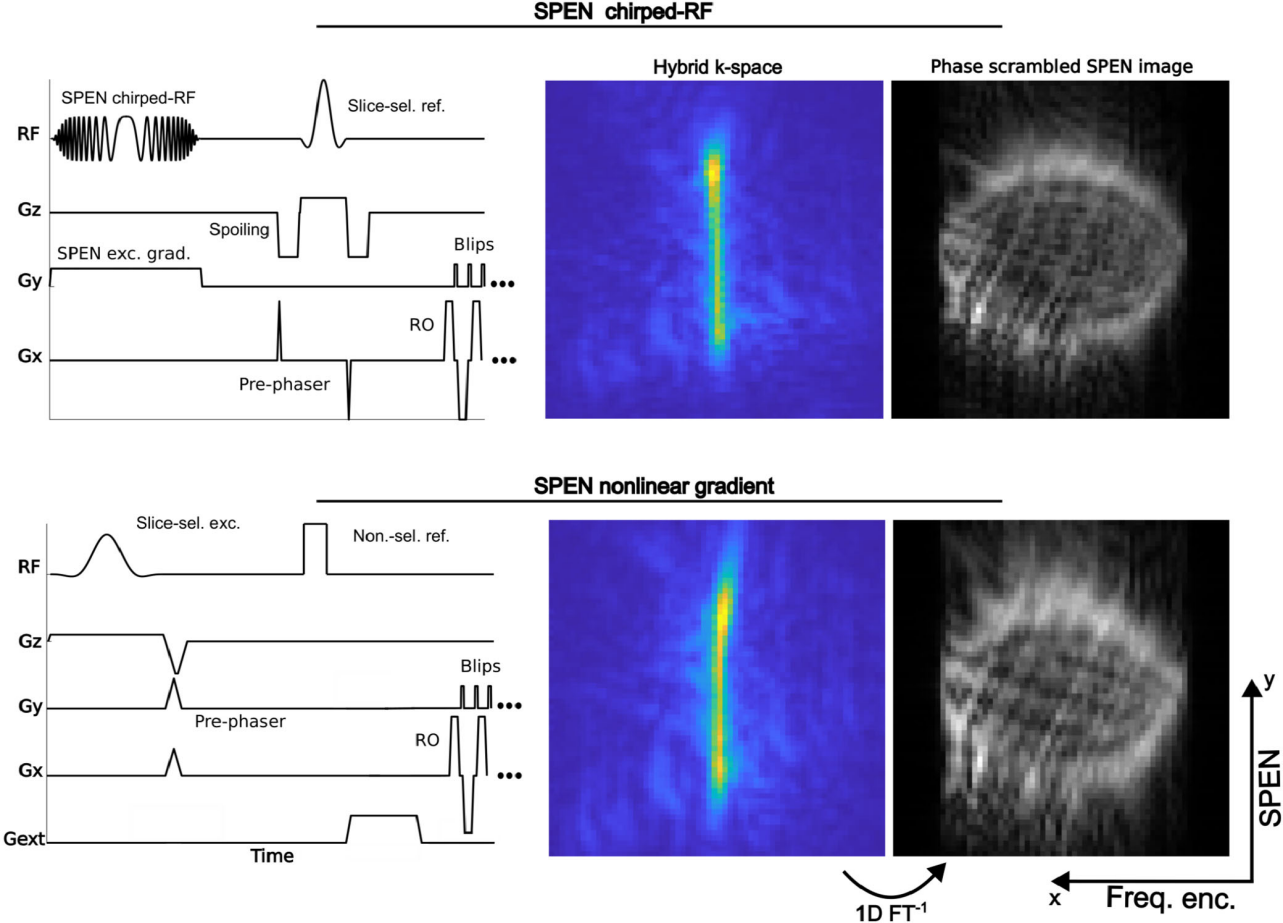
SPEN‐SE‐EPI experiment comparison. For the SPEN‐SE‐EPI sequence shown in the top row, the Open‐SPEN Pulseq implementation was used. We designed a 90°‐chirped‐RF pulse with a duration of 4 ms, a sweeping bandwidth of 50 kHz (TBW = 200), an amplitude modulation factor of 40 and an excitation gradient‐strength of $250\;\frac{\text{kHz}}{m}$ was used. Slice‐selective 180° refocusing was used. The SPEN gradients were calculated by the Open‐SPEN implementation to excite an FOV of 220 mm. In the bottom row, the SPEN‐SE‐EPI sequence using the external gradient method is shown. A slice‐selective 90°‐sinc excitation pulse was used with non‐selective 180° refocusing. The quadratic gradient event was placed between the refocusing pulse and the first readout, exploiting the delay to achieve the desired TE. For both SPEN methods, a matrix size of 64×64 and a TE = 60 ms were used, in combination with a conventional EPI readout scheme. The horizontal direction was frequency‐encoded, and SPEN was applied to the vertical direction. Every second line of the hybrid *k*‐space of both methods (middle column) was flipped from left to right and aligned to the odd readouts by linear phase modulation. The blurred SPEN images (right column) were obtained by an inverse Fourier transform in the horizontal frequency‐encoding direction.

To extend this method to imaging, we designed a SPEN‐SE‐EPI sequence in Pulseq [4, 12, 15, 16, 20, 27, 28] and compared it to a conventional Fourier‐encoded SE‐EPI sequence under inhomogeneous magnetic field conditions. Figure 5 shows a schematic of the SPEN‐SE‐EPI sequence with the chirped‐RF pulse.

### 3.2 SPEN With Additional Gradient Hardware

For the gradient hardware approach, we used a custom‐built 84‐channel matrix gradient coil capable of generating arbitrary SEMs [23, 25] within physical constraints. This coil can create a quadratic SEM and the corresponding quadratic phase profile. The system was powered by 12 additional gradient power amplifiers controlled by custom electronics [29]. The MRI system clock signal synchronized the additional gradient hardware with the MRI system.

We grouped multiple coil elements into 12 clustered channels and optimized their initial weights to achieve the desired quadratic SEM. A gradient amplitude of $313\;\frac{\text{mT}}{m^{2}}$ and a duration of 5 ms was selected to produce a smooth phase profile. We verified the accuracy of the generated magnetic field using a gradient‐echo‐based B 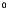
‐field mapping sequence implemented in Pulseq.

The one‐dimensional spin‐density readout experiment was repeated using the external quadratic gradient instead of the chirped‐RF pulse, demonstrating the feasibility of SPEN with external gradients. Figure 4 shows the corresponding sequence.

To further assess imaging capabilities, we designed a SPEN‐SE‐EPI sequence in Pulseq that incorporates an external gradient event (Figure 5). This implementation used the delay necessary for achieving the desired TE as a placeholder for the external gradient event. We subsequently performed a SPEN‐SE‐EPI experiment using the additional gradient hardware and compared the results with those from the Fourier‐encoded SE‐EPI and the Open‐SPEN chirped‐RF pulse methods.

SPEN data were reconstructed using a convolution‐based method [30, 31], applying the Fresnel transform for the SPEN direction and an inverse Fourier transform for the frequency‐encoding direction. Further details on SPEN reconstruction and aliasing suppression are described in the literature [21].

All imaging experiments were performed on a 3T Siemens Trio MRI system (Siemens Healthineers, Erlangen, Germany), equipped with a custom‐built 31‐channel RF head coil for signal acquisition and a single‐channel circularly polarized transmit coil for excitation. Both coils were designed to fit within the matrix gradient coil. To assess the methods' robustness against magnetic field inhomogeneities, we scanned a spherical phantom containing rods of different diameters under intentionally poor $B_{0}$ field shimming conditions.

## 4 Results

### 4.1 Chirped‐RF Implementation

An example of the real part of a chirped‐RF pulse is shown in Figure 4a. SPEN excitation and acquisition gradients are designed directly from the imaging parameters, while respecting system limitations and the SPEN condition.

Simulation results of the chirped‐RF pulse (Figure 3a) demonstrate that the bandwidth‐corrected pulse produces a homogeneous flip angle across the frequency offset, underlining its robustness against off‐resonance effects. The corresponding excitation profile is steep and accurately matches the intended bandwidth. The simulated one‐dimensional phase profile is shown in Figure 3b. A quadratic phase profile is accumulated and centered at one edge of the target FOV after excitation. During acquisition, the application of gradients shifts the vertex of the phase parabola back across the FOV.

Experimental one‐dimensional spin‐density results acquired with MRI are presented in Figure 4. Using the chirped‐RF pulse together with the designed gradient scheme, the signal is confined to the parabola vertex during acquisition. Reconstruction of the spin density is then achieved solely through deconvolution that compensates for the quadratic phase, unfolding the scrambled phase of the acquired signal, without requiring a Fourier transform [1].

**FIGURE 6:**
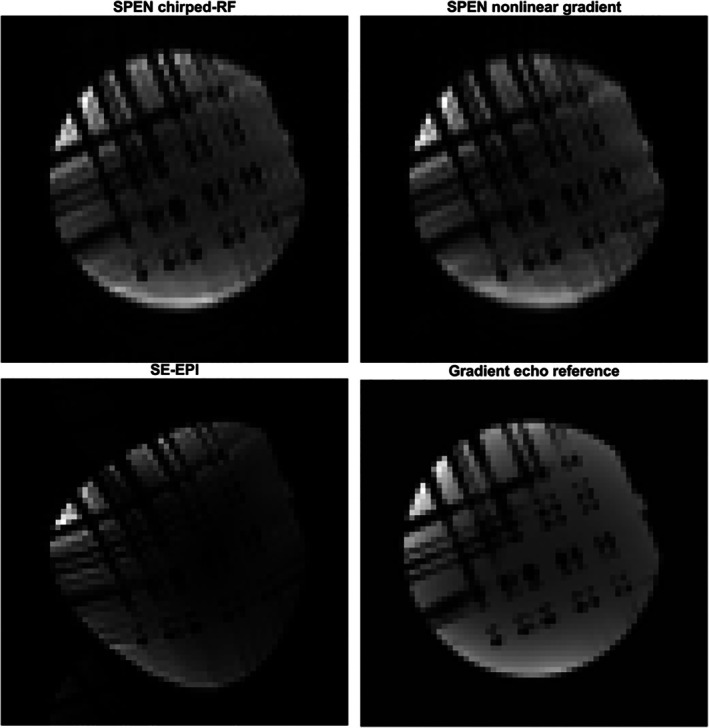
Comparison of the reconstructed SPEN images. The image on the bottom left shows a Fourier‐encoded SE‐EPI image for comparison. Given that intentionally poor shimming was carried out to assess the robustness of the implemented SPEN methods against magnetic field inhomogeneities, the SE‐EPI image appears distorted. The chirped‐RF SPEN image is shown on the upper left and the external gradient SPEN image upper right. The SE‐EPI image was reconstructed by applying a two‐dimensional inverse Fourier transform on the raw data. Both SPEN images were reconstructed by applying an inverse Fourier transform in the frequency encoding direction and a convolution‐based reconstruction in the SPEN direction. The GRE image shown on the bottom right serves as a reference. All images were obtained in succession with the same shim settings and the SE‐EPI images (SPEN & conventional) with the same EPI readout.

Two‐dimensional experiments further demonstrate that Open‐SPEN can encode along arbitrary directions. In the SPEN‐SE‐EPI sequence shown in Figure 5, SPEN was applied along the former phase‐encoding direction to counteract the low bandwidth and associated geometric distortions. For each frequency‐encoding readout, the signal contribution remains confined in the SPEN direction, producing a vertical high‐intensity line in hybrid *k*‐space. After applying an inverse Fourier transform along the frequency‐encoding direction, the characteristic blurred SPEN image is obtained (Figure 5, right column). The quadratic phase present during readout is convolved with the acquired signal, resulting in phase scrambling in the SPEN direction. A convolution‐based reconstruction removes this effect and produces the undistorted phantom image shown in Figure 6c, with only minor ringing and low‐level ghosting caused by hardware imperfections.

### 4.2 SPEN With Additional Gradient Hardware

**FIGURE 7:**
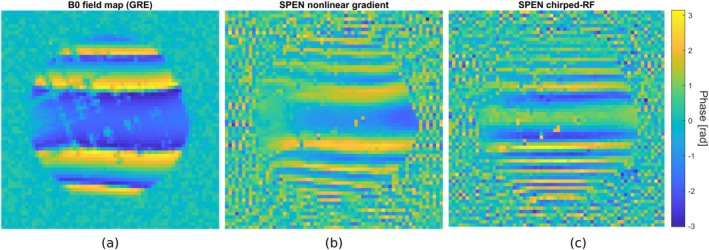
Quadratic phase profiles used for SPEN. A GRE‐based B0‐mapping sequence was used in (a) to visualize the characterized quadratic phase profile using the external matrix gradient coil. Given that the quadratic phase profile cannot be removed by the linear acquisition gradients, the phase of the acquired SPEN images from Figure 6 represents the actual achieved quadratic phase profiles. (b) Shows the phase profile of the external gradient SPEN method. (c) shows the achieved phase profile using the Open‐SPEN chirped‐RF method. The phase in radians is displayed color‐encoded.

The quadratic phase profile generated by the matrix gradient coil is shown in Figure 7a, acquired with a gradient‐echo field mapping sequence. The profile appears homogeneous, with only small deviations near the phantom edges. When applied directly before acquisition in the one‐dimensional spin‐density sequence (Figure 4b), the signal contribution becomes confined to the parabola vertex, blurring the *k*‐space data. The resulting phase‐unscrambled signal is similar to that of the chirped‐RF method, with slight deviations attributed to imperfections in the gradient coil, amplifier, or field characterization. This experiment shows that SPEN with nonlinear gradient hardware is feasible without the need for a chirped‐RF pulse.

Incorporating this phase profile generation method into an SE‐EPI sequence (Figure 5, left column) yields the hybrid *k*‐space shown in the center column, where the vertical SPEN line confirms confinement of the signal to the vertex during acquisition. The reconstructed image (Figure 6b), obtained with the same method as for the chirped‐RF case, shows a distortion artifact‐free phantom. These results demonstrate the feasibility of extending the nonlinear gradient approach to two‐dimensional SPEN.

### 4.3 Comparison of Chirped‐RF and Nonlinear Gradient SPEN Methods

A direct comparison of the hybrid *k*‐space data from both methods is shown in Figure 5 (middle column). The nonlinear gradient method produces a more homogeneous, high‐intensity line, while both lines extend over a similar range and reflect comparable phase profiles and encoding properties. Gradient delay imperfections cause phase shifts in the frequency‐encoding direction. These shifts are visible as a saw‐tooth pattern in hybrid *k*‐space and can not always be corrected by the self‐navigated nature of SPEN. Thanks to direct spatial encoding in the former phase‐encoding direction, the gradient‐delay‐induced phase shift can in theory be corrected by simply aligning the hybrid *k*‐space lines to a straight, high‐intensity line. This is limited to a minimal shift of one pixel. In the case of the nonlinear gradient method, the phase shift was incidentally aligned to the sample raster, enabling effective compensation of the phase shift. Residual phase shifts result in low‐level ghosting in the final image, as is visible with the chirped‐RF pulse method.

After Fourier transform, both methods yield blurred SPEN images (Figure 5, right column). Blurring is stronger for the nonlinear gradient method, consistent with the slightly lower steepness of its quadratic phase, as confirmed in Figure 7. The chirped‐RF phase parabola is steeper and more homogeneous, producing a less blurred image due to a smaller convolution area. Importantly, this difference is not visible in the phantom image (Figure 6) when it is accounted for during reconstruction. Moreover, the steepness of the quadratic phase can be tuned by adjusting the gradient strength or duration, depending on sequence timing constraints.

The reconstructed images (Figure 6), obtained by Fourier transform in the frequency‐encoding direction and deconvolution in the SPEN direction, are shown alongside a SE‐EPI reference. Both SPEN methods provide undistorted phantom images with quality comparable to the reference, while the SE‐EPI image suffers from severe distortions and system imperfection induced ghosting. In contrast, ghosting is minor for the chirped RF method and absent for the nonlinear gradient method due to the self‐navigated nature of SPEN. However, phase shifts that are not aligned to the sample raster cannot be corrected and produce minor ghosting, as with the chirped‐RF method. The total sequence duration of the chirped‐RF approach is about 110 ms, owing to the long RF pulse and additional gradient events. By comparison, both SE‐EPI and nonlinear gradient SPEN last 90 ms, benefiting from a short sinc‐excitation pulse and reduced TE.

In our experiments, the RF energy, defined as the time integral of the squared RF pulse amplitude (${\text{Hz}}^{2}$), was much lower for the sinc‐pulse used in the quadratic gradient implementation compared to the chirped‐RF pulse, as shown in Table 1. When converted to RF power by dividing the pulse energy by its duration, this corresponds to roughly six times lower relative SAR for the quadratic gradient method, since SAR $\propto {\left|B_{1}\right|}^{2}$ (Table 1).

**TABLE 1:** The relative energy of the RF pulses is expressed in the units of RF amplitude squared and multiplied by time (Hz). The RF amplitude can be converted to Tesla (T) by dividing the resulting value by the gyromagnetic ratio (γ). Similarly, the power can be converted to $T^{2}\cdot s$ by dividing the given value in Hz by $\gamma^{2}$, prior to taking the integral. Nonetheless, the absolute SAR is subject dependent. For comparison purposes, a bandwidth of 50 kHz and a duration of 4 ms were used for the 90°‐chirped‐RF pulse for reasonable robustness towards off‐resonance effects. A 90°‐sinc pulse with a duration of 3 ms and a TBW of 4 was used for the quadratic gradient method.

Relative RF power comparison		
	Chirped‐RF pulse	Quadratic gradient
RF energy [Hz]	3300	65
RF peak power [Hz 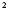 ]	$1\times 10^{6}$	$1.10\times 10^{5}$
RMS B 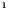 amplitude [Hz]	915	147

## 5 Discussion

Our results provide the first practical validation that the quadratic phase profile required for SPEN can be generated using nonlinear gradient hardware, thereby confirming the theoretical equivalence to the conventional chirped‐RF pulse method. This finding has important implications for the future development and clinical translation of SPEN imaging.

The main advantage of the nonlinear gradient approach is that it circumvents SAR limitations. In the conventional method, achieving a high quadratic phase coefficient β—and thus increased robustness—requires long‐duration, high‐bandwidth chirped‐RF pulses, which lead to substantial SAR deposition [32]. In contrast, our nonlinear gradient implementation decouples the generation of the quadratic phase from the RF pulse itself. As a result, one of the major obstacles for SPEN at high field strengths or in SAR‐intensive MS protocols is effectively eliminated.

In addition, the nonlinear gradient method allows for a more flexible sequence design. Because it avoids the long RF durations inherent to chirped‐RF pulses, it enables shorter TEs. As the quadratic phase profile is time‐independent, placing the external gradient event before rather than after the refocusing pulse as shown in Figure 5 would result in an even greater reduction in TE, since the phase parabola would simply be inverted by the refocusing pulse. However, the inverted convolution kernel must be taken into account during the reconstruction process. This capability directly facilitates efficient MS imaging, which has long been a challenge for conventional SPEN. Extending chirped‐RF SPEN to MS typically forces severe trade‐offs: Either multiple high‐SAR chirp pulses, inefficient volumetric excitation schemes that prolong overall scan time, or the use of complex, long‐duration 2D RF pulses that increase the minimum TE and are limited by gradient system performance [4, 13, 33, 34, 35, 36]. By enabling true slice‐selective excitation and refocusing without incurring any of these penalties, our nonlinear gradient approach makes robust three‐dimensional SPEN applications far more feasible. A MS version of a SPEN‐SE‐EPI sequence with nonlinear gradients would only require the incorporation of an external SPEN‐gradient event into a conventional slice‐selective SE‐EPI sequence. This is because adding the external SPEN‐gradient event itself does not interfere with the rest of the sequence.

Our nonlinear gradient method, however, requires specialized nonlinear gradient hardware, which is currently unavailable on clinical MRI systems. Its simplicity and efficiency could be substantially improved if the quadratic phase profile was generated by a dedicated gradient coil rather than by a complex matrix gradient system [7, 24, 26]. Such dedicated hardware would be especially beneficial for ultrafast imaging of large volumes or in regions that are difficult to shim, for example off‐isocenter areas such as the distal extremities, or in situations where high spatial resolution is needed. In these scenarios, a dedicated coil could improve image quality without extending acquisition time. By contrast, our open‐source chirped‐RF implementation requires no additional hardware and can be deployed on scanners of several vendors, which makes it highly attractive for low‐field applications where SAR is of less concern.

Finally, it is important to emphasize that the chirped‐RF method also offers unique capabilities that are not replicated by the nonlinear gradient‐based approach. One prominent example is the “full‐refocusing” mode, which provides enhanced robustness against off‐resonance effects [4, 16]. This remains an exclusive feature of the chirped‐RF technique. Nevertheless, for many practical applications—especially those requiring high resolution, short TE, or three‐dimensional MS coverage at clinical field strengths, the advantages of the nonlinear gradient approach are compelling and open up new opportunities for the advancement of SPEN imaging.

## 6 Conclusions

We established theoretically and demonstrated experimentally that additional nonlinear gradient hardware can be utilized for SPEN by generating the required quadratic phase profile externally. The proposed approach produces high‐quality SPEN‐SE‐EPI images, comparable to the conventional chirped‐RF method, while fundamentally overcoming its constraints related to SAR and prolonged RF durations. By enabling slice‐selective excitation and shorter TEs, the nonlinear gradient SPEN method paves the way for robust three‐dimensional SPEN applications. This significantly enhances its potential for clinical translation, especially since the in vivo capabilities of conventional SPEN have already been demonstrated [27] and quadratic magnetic fields have already been used for other in vivo imaging purposes [37]. A comprehensive study of MS SPEN‐SE‐EPI with nonlinear gradients is the subject of future work. To foster continued research and ensure reproducibility, we provide an open source implementation of the conventional chirped‐RF SPEN method in Pulseq, along with an educational Bloch simulation script for modeling the SPEN forward operator (https://github.com/andih98/Open‐SPEN.git). The implementation for the control of external gradient hardware with Pulseq is available upon request.

## Funding

This work was partially supported by the Carl Zeiss Foundation (CZS Wildcard Project MINI, Grant No. P2023‐03‐016).

## Data Availability

The data that support the findings of this study are openly available in Open‐SPEN at https://github.com/andih98/Open‐SPEN.git.
